# Somatostatin as an Active Substance in the Mammalian Enteric Nervous System

**DOI:** 10.3390/ijms20184461

**Published:** 2019-09-10

**Authors:** Slawomir Gonkowski, Liliana Rytel

**Affiliations:** 1 Department of Clinical Physiology, Faculty of Veterinary Medicine, University of Warmia and Mazury in Olsztyn, Oczapowski Str. 13, 10–718 Olsztyn, Poland; 2 Department and Clinic of Internal Diseases, Faculty of Veterinary Medicine, University of Warmia and Mazury in Olsztyn, Oczapowski Str. 14, 10–718 Olsztyn, Poland

**Keywords:** somatostatin, enteric nervous system, intestinal motility, gastrointestinal tract

## Abstract

Somatostatin (SOM) is an active substance which most commonly occurs in endocrine cells, as well as in the central and peripheral nervous system. One of the parts of the nervous system where the presence of SOM has been confirmed is the enteric nervous system (ENS), located in the wall of the gastrointestinal (GI) tract. It regulates most of the functions of the stomach and intestine and it is characterized by complex organization and a high degree of independence from the central nervous system. SOM has been described in the ENS of numerous mammal species and its main functions in the GI tract are connected with the inhibition of the intestinal motility and secretory activity. Moreover, SOM participates in sensory and pain stimuli conduction, modulation of the release of other neuronal factors, and regulation of blood flow in the intestinal vessels. This peptide is also involved in the pathological processes in the GI tract and is known as an anti-inflammatory agent. This paper, which focuses primarily on the distribution of SOM in the ENS and extrinsic intestinal innervation in various mammalian species, is a review of studies concerning this issue published from 1973 to the present.

## 1. Introduction

Somatostatin (SOM) was isolated for the first time from the ovine hypothalamus in 1973 as a factor which inhibits growth hormone release [1]. It is a cyclic oligopeptide, which in mammals occurs in two forms: shorter–built of 14 amino acids and longer–composed of 28 amino acids [1,2]. SOM is produced in various organs and is known as a strong inhibitory factor. It acts via five types of G protein-coupled somatostatin receptors (from SSTR1 to SSTR5, with two isoforms of SSTR2: SSTR2A and SSTR2B), but within the enteric nervous system (ENS), mainly SSTR1 and SSTR2 have been observed [3]. Previous studies have reported that most SOM in the mammalian body is localized in the gastrointestinal (GI) tract and pancreas [4,5]. However, it has been shown that about 90% of SOM in the GI tract is situated in the gastrointestinal endocrine cells, and only 10% is located in the nervous structures [6]. In spite of the relatively small amount of SOM produced by the enteric neurons, this substance plays important functions in this part of the nervous system, both under physiological conditions and during pathological states. The review of the literature primarily concerning the distribution of SOM in the ENS and extrinsic innervation of the GI tract in various mammal species is set out below.

## 2. Organization of the Enteric Nervous System (ENS)

The ENS is a part of the autonomic innervation of internal organs. It is localized in the wall of the GI tract from the oesophagus to the rectum. Since the ENS is characterized by complex organization, a high number of nervous structures and a high level of independence from the central nervous system, it is often called “the second brain” or “the intestinal brain” [7,8,9]. The ENS is composed of millions of neuronal cells, making it the second (after the brain, but before the spinal cord) largest nervous structure in the living organism in terms of the number of neurons [7,8]. 

**Figure 1:**
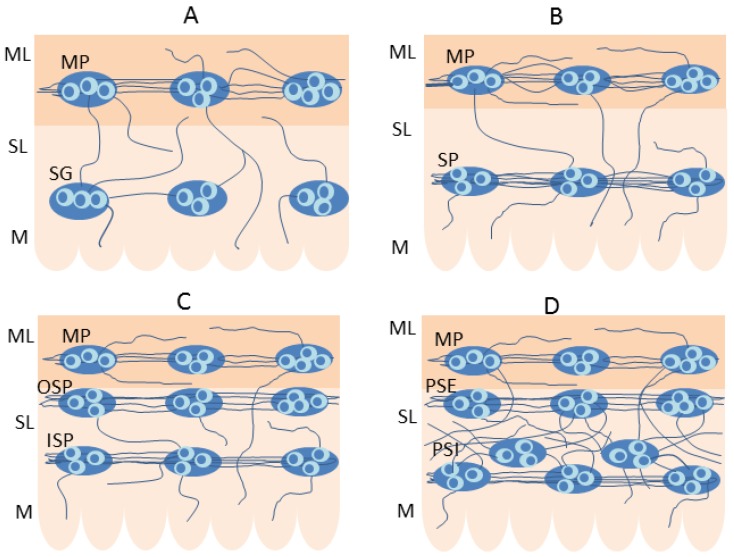
The organization of the enteric nervous system in various mammal species: **A**–oesophagus and stomach of the vast majority of mammal species, **B**–small and large intestine of rodents, **C**–small and large intestine of the domestic pig, **D**–human small and large intestine. ML = muscular layer, SL = submucosal layer, M = mucosal layer, MP = myenteric plexus, SG = submucous ganglia, SP = submucous plexus, OSP = outer submucous plexus, ISP = inner submucous plexus, PSE = plexus submucosus externus, PSI = plexus submucosus internus

The enteric neurons are grouped in the intramural ganglia, which are connected to each other by a dense network of nerve fibres. The number and exact localization of these ganglia clearly depend on the mammalian species and the segment of the GI tract (Figure 1). In small mammals (for example in rodents), two kinds of intramural ganglia are observed in the gastrointestinal tract. These are the myenteric ganglion, located between longitudinal and circular muscle layers, and submucous ganglion, located near the lamina propria of the mucosal layer [10]. The myenteric ganglia, connected to each other by the dense nerve fibres, form the myenteric plexus (MP) in the whole GI tract (Figure 1A,B). In turn, the nerve fibres between the submucous ganglia in the oesophagus and stomach (Figure 1A) are not dense and, therefore, in this part of the GI tract there is no submucous plexus. Contrary to the above-mentioned segments of the GI tract, the submucous plexus is present in the small and large intestines of rodents (Figure 1B).

In large mammals (for example, in the domestic pig), the ENS in the oesophagus and stomach is similar to rodents and consists of the myenteric plexus and separate submucous ganglia [11,12]. The only exception is the forestomach in the ruminants, where only the mucosal plexus is present [13]. 

Instead, in the small and large intestine of large mammals, apart from the myenteric plexus located (like in rodents) between longitudinal and circular muscle layers, two types of submucous plexuses (Figure 1C) can be identified [9]. The first is the outer submucous plexus (OSP) located near the internal side of the circular muscle layer, and the second is the inner submucous plexus (ISP), positioned (like the submucous plexus in rodents) near the lamina propria of the mucosal layer. Two kinds of submucous enteric plexuses, known as plexus submucosus externus (PSE) and plexus submucosus internus (PSI) are also observed in the human small and large intestine [14]. The first is located like the OSP in the domestic pig, and the second is (contrary to the pig) multi-layered and located at a different depth within the inner part of the submucous layer (Figure 1D) [14].

The enteric neurons are very different in terms of morphological properties, functions and electrophysiological characteristics, but the greatest differentiation of them concerns the neurochemical characterization, i.e., the abilities of particular neuronal cells to synthesize various active substances. Previous studies have described that enteric neurons may synthesize several dozen active factors, which act as neuromediators and/or neuromodulators, intracellular transporters and enzymes. The most important of them (apart from acetylcholine–the main neuromediator within the ENS) include, among others, vasoactive intestinal polypeptide (VIP), galanin (GAL), nitric oxide, substance P (SP), calcitonin gene-related peptide (CGRP) and serotonin [8,9,10,15]. One of the neuronal factors which is present in the ENS is also somatostatin (SOM).

## 3. The Presence of Somatostatin (SOM) in the Enteric Neurons in Particular Mammal Species

To date, the presence of SOM has been described in the ENS in various mammal species, including humans [16,17,18,19]. It should be noted that clear interspecies differences in the exact distribution of this peptide in the ENS have been observed [19]. Moreover, the distribution of SOM differs in various segments of the GI tract in the same species, and often even observations concerning the same fragments of the GI tract in one species performed by different authors were not the same [17,20,21,22]. The latter differences may result from the fact that the concentration of SOM in tissues clearly depends on the manner of preparation of tissues to study. For example, longer storage of fixed tissues causes a decrease in the SOM concentration of up to 40% of its original value [23]. Moreover, the ENS may undergo various stimuli, such as the influence of gut microbiota, changes in diet or environmental factors [24,25]. These stimuli probably may also change the concentration of SOM in the enteric nervous structures. The distribution of SOM in the enteric nervous system in the particular mammal species is set out below.

### 3.1. Guinea Pig

Soon after the first description of SOM in the hypothalamus, it was also observed in the nervous structures of the ENS. One of the first publications concerning this issue described the presence of SOM within the MP of the GI tract of guinea pig, which is the best known species in terms of the distribution and functions of SOM in the ENS [26]. In this publication SOM was observed in intraganglionic neurons and nerve fibres located within the myenteric and submucous plexus of the ileum [26]. Nerves containing SOM were often closely related to the enteric neurons and formed a basket-like arrangement around neurons, which suggests that SOM is present in the interneurons of the ENS. Further studies confirmed the occurrence of SOM in the guinea pig ENS and showed that neuronal cells containing this peptide in the small intestine amount to about 17% of all enteric neurons in the MP and about 5% of total neuronal population in the submucous plexus [27,28,29]. SOM was also observed in the intramuscular and intramucosal nerve fibres located in the small intestine [26,27]. In the mucosal layer, such nerves are relatively dense and form small bundles [27]. 

**Figure 2:**
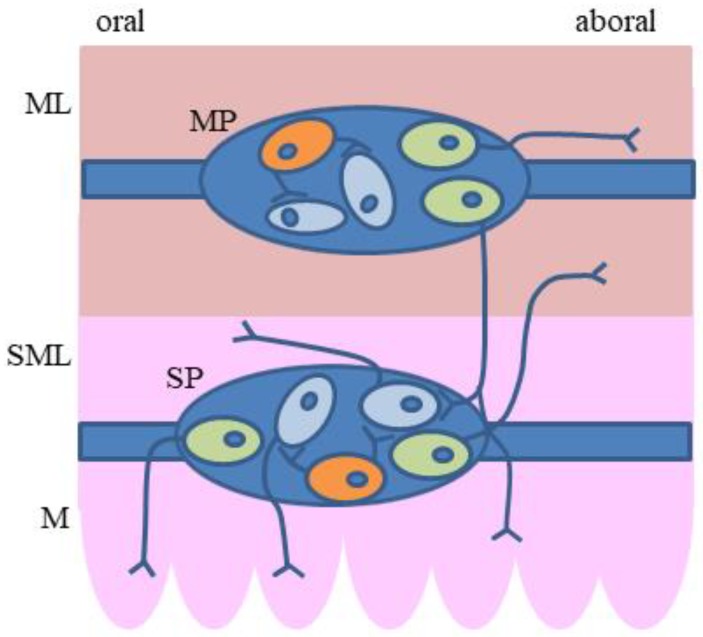
The distribution of two main types of somatostatin (SOM)-positive enteric neurons (secretomotor neurons and interneurons) in the guinea pig intestine: green–SOM-positive secretomotor neurons, orange–SOM-positive interneurons, blue–other neurons, ML = muscular layer, SML = submucosal layer, M = mucosal layer, MP = myenteric plexus, SP = submucous plexus.

Previous studies have also shown that SOM may occur in various types of guinea pig enteric neurons (Figure 2). 

Studies of various authors often differ regarding the exact distribution of SOM in the enteric neurons in the guinea pig small intestine. According to Hu et al., SOM has been observed in neurons belonging to group II of the Dogiel classification and is characterized by large, elongated or round perikaryon with a single long and thick axon projected in the aboral direction [16]. In turn, other studies have shown that SOM occurs in two types of neurons located in the ENS of guinea pig small intestine: large neurons, which were usually found in groups, and a small population of secretomotor neurons, in which neuropeptide Y was also noted [10,30,31]. Similar observations have been performed by Furness et al., who stated that SOM is present in secretomotor, cholinergic neurons located in the submucous plexus of the guinea pig small intestine [32,33]. These neurons not only contain acetylcholine, but may also show immunoreactivity to a wide range of other active substances, such as cholecystokinin, neuropeptide Y, calcitonin gene-related peptide, dynorphin and very often galanin [33]. Moreover, Furness et al. found that neurons containing SOM in the myenteric and submucous plexus of the guinea pig small intestine are similar [32]. In both kinds of enteric plexuses, SOM-positive neuronal cells belong to the type-III Dogiel cells and were characterized by from 2 to 10 thin, branched dendrites and one long axon. The axons of these neurons located in the myenteric plexus supply not only intestinal smooth muscles, but also ran to the submucosal layer and the mucosa. In turn, neurons located within the submucous plexus have processes innervating the mucosal layer [32]. 

Other studies have shown that SOM-positive neurons in the guinea pig small intestine are relatively numerous in the submucous plexus and amounted to about 30% of all neuronal cells [34]. Such neuronal cells are mainly located in peripheral parts of the ganglia and, first of all, innervate the bases of the villi in the mucosa, and do not supply the myenteric plexus or other ganglia in the submucous plexus [34]. Moreover, it is known that in terms of electrophysiological properties, neuronal cells containing SOM are characterized by the occurrence of fast excitatory inputs with multi-large amplitude or (very rare) slow excitatory inputs and the absence of inhibitory inputs [34]. The presence of SOM in two kinds of neurons (secretomotor neurons and interneurons) in the enteric plexuses located in the guinea pig small intestine has been confirmed by more recent studies [29].

SOM has also been detected in the enteric nervous structures of other parts of the guinea pig digestive tract but, contrary to the small intestine, knowledge concerning this issue is more fragmentary. For the large intestine, it is known that the number of SOM-positive neuronal cells in the colonic enteric plexuses is generally similar to that observed in the small intestine [27]. Nevertheless, contrary to the ileum, within the colon, SOM-positive nerves do not supply the mucosal layer, and such nerves in the myenteric plexus are denser and have good visible varicosities [27]. In turn, Probert et al. did not observe SOM in the neurons located in the myenteric plexus of the guinea pig colon, but they have described the intraganglionic SOM-positive nerves, which belong to separate subpopulations of non-cholinergic and non-adrenergic p-type nerves [35]. Such nerves were rather infrequent, but were characterized by relatively large varicosities. Similar observations were performed by Leander et al., who also did not observe SOM-positive neurons in the MP of the guinea pig colon and noted that nerves immunoreactive to SOM located both in the myenteric plexus as well as in the colonic muscular layer were very sparse [36]. Other studies have described SOM-positive neuronal cells both in the myenteric and the submucous plexus in the large intestine of the guinea pig, although myenteric neurons immunoreactive to SOM were only visible after colchicine and veratridine treatment or after myotomy operation [37]. Such neurons were round or oval and were characterized by oral projection, contrary to the ileum, where SOM-positive myenteric neurons show caudal projection [37]. Moreover, Messenger and Furness described SOM-positive colonic intramuscular nerves, which grouped together and formed bundles [37]. Similar results were obtained by more recent studies, in which the number of SOM-positive enteric neurons in the guinea pig GI tract in freshly-fixed preparations was minimal and increased after colchicine treatment [16]. In the same studies, numerous nerves containing SOM were described in the myenteric ganglia and inter-ganglionic fibre tracts.

Knowledge of the distribution of SOM-positive neuronal cells and nerves in the wall of the guinea pig stomach is more fragmentary. SOM has been observed in about 6% of all neuronal cells located in the myenteric plexus [38]. The same authors also described SOM-positive nerves in the gastric muscular layer and in the myenteric plexus. Other studies have shown that neurons immunoreactive to SOM are primarily located in the myenteric ganglia positioned at the lesser curvature of the stomach [39]. It is also known that SOM in the enteric neurons located in the stomach may co-localize with other active substances, including substance P, acetylcholine, calretinin, calbindin and enkephalin [38,40,41]. The majority of SOM-positive neurons located in the gastric myenteric plexus also contain substance P (about 85% of all SOM-positive neurons) and/or enkephalin (above 60% of all SOM-positive neurons) [41]. The projections of SOM-positive neuronal cells in the cardiac myenteric plexus are not clear. Some authors have noted that these neurons (apart from the muscular layer) supply the gastric mucosa, while others have not observed such projections [40,41].

### 3.2. Domestic Pig

In recent years, the domestic pig has increasingly been used as an animal model during studies concerning the influence of various pathological states on the GI tract and the ENS. This is because considerable similarities in the organization, neurochemistry and electrophysiology between human and porcine ENS have been described [42,43]. Therefore, it can be concluded that the reactions of the enteric neurons on pathological or toxic stimuli in the pig may be helpful for a clearer understanding of these mechanisms in the human body.

Generally, SOM in the porcine ENS is present in the same types of the enteric neurons as in the guinea pig. Namely, the main two classes of the porcine enteric neurons, in which SOM is observed are 1) type-V neurons located in myenteric and outer submucous plexus (not in the inner submucous plexus) playing the role of the descending interneurons and 2) type-IV neurons observed in all types of intramural plexuses and playing the role of secretomotor neurons [42,44,45]. It has been shown that in the MP of the porcine ileum, SOM is present in above 90% of all type-V neurons, in which it may co-localize mainly with CGRP [44]. In turn, SOM-positive type-IV neurons do not contain CGRP. In the OSP, type-V neurons have been noted as single cells or as aggregates of some perikarya, and about 60% of them displayed co-localization of SOM and CGRP [46].

In previous studies, clear differences in the localization of SOM-positive nervous structures have been observed between the particular segments of the porcine GI tract. The most is known about the distribution of SOM-positive enteric nervous structures in the small and large intestine. The number of enteric neuronal cells in these GI tract segments is rather low. The exact number depends on the fragment of the intestine and are different in various studies. According to Gonkowski and Calka, in the porcine descending colon, the number of SOM—immunoreactive neuronal cells in the MP, and OSP amounts to about 2% of all neurons and in the ISP, it oscillates about 4% [17]. In turn, in the ascending colon, SOM has been noted in about 5% of the total neuronal population in all types of the enteric plexus [47]. Petto et al. usually observed one SOM-positive neuron per ganglion in the case of MP and ISP and about 3 neurons immunoreactive to SOM per ganglion in the OSP [48]. Generally, they showed the presence of SOM in less than 1% of all neurons in the MP and ISP and in above 4% in the OSP. In the small intestine (ileum), SOM was noted in 7% of all neurons in the MP, 3% in the OSP, and 17% in the ISP [20]. Moreover, both in the large and small intestine, the relatively rare SOM-positive nerve fibres were noted in the muscular and mucosal layers, as well as inside the ganglia within all types of the enteric plexuses [17,20,47,48,49,50]. The only exception was the myenteric plexus in the descending colon, where SOM-positive nerves formed a dense network [17]. Knowledge of the distribution of SOM in the enteric nervous structures in the porcine stomach and oesophagus is more scarce. In the stomach, SOM has been described in single neurons located in the submucous ganglia and rare nerves in the muscular and mucosal layers, but not in the cells within the myenteric plexus [51]. In the oesophagus, SOM has been described in rare, delicate nerves located in the muscular and mucosal layers [52]. 

### 3.3. Human

To date, SOM has been detected in the enteric nervous structures in various segments of the human digestive tract [18,53,54,55,56,57]. In the large intestine, in spite of the fact that older studies have not described the enteric neuronal cells containing SOM [54], such cells are present in the MP, PSE and PSI [53,57]. The number of SOM-positive neurons is aligned in all types of the enteric plexus and amounts to about 30% of the total neuronal population [53]. In turn, the observation of the number of SOM-positive cells in the submucous plexuses of the human large and small intestine performed by Kustermann et al. is slightly different [18]. Those authors found that the percentage of neurons containing SOM in the PSE amounts to 29% of all neuronal cells in the large intestine and 4% in the small intestine. These values in the PSI were 40% and 13%, respectively. Moreover, extreme differences in the number of SOM in the enteric neurons were observed between the particular patients included in the study. The percentage (in respect to total neuronal population) of neuronal cells immunoreactive to SOM ranged from 0% to 58% in the PSE and from 0% to 59% in the PSI [18]. In the human intestine, SOM has been detected in pseudo-uni- or multi-axonal-non-dendritic Dogiel type-II neurons, which in the human ENS (similar to the guinea pig ENS) probably belong to the intrinsic primary sensory neurons (IPAN) and have long axons innervating the submucosal and mucosal layers [18,58]. In these neurons, located in the MP, SOM co-localizes with calretinin (such co-localization has been noted in 89.6% of all type-II neurons) and/or with substance P (in 86.6% of all type II neurons) [58]. In the MP of the human intestine, SOM has also been detected in Dogiel V-type neurons, which probably play a role as descending interneurons, as well as in Dogiel III-type neurons—a small population of uni-axonal cells with long and slender dendrites, whose exact functions are unknown [58]. Contrary to the myenteric plexus, in the submucous plexuses of the human intestine, neurons simultaneously showing the presence of SOM and calretinin are rare [18]. Other studies have shown the co-localization of calbindin and SOM in the above-described type-III neurons [57]. The degree of co-localization clearly depended on the type of the enteric plexus. It was negligible in the myenteric plexus (SOM was present in 1.7% of all myenteric calbindin–positive neurons in the small intestine and in 4.6% in the large intestine), and very high in submucous plexuses (about 80% in the small intestine and about 80% in the large intestine) [57]. Moreover, SOM-positive nerve fibres have been observed in both the small and large human intestine. Such fibres are located in the muscular and mucosal layers, as well as inside the enteric ganglia [18,53,54]. Nerves containing SOM were denser in the mucosa than within the muscular layer [57]. Such nerves were relatively abundant near the blood vessels located in the intestinal wall, contrary to vessels in the mesentery, near which SOM-positive nerves were rather rare [59].

Knowledge of the distribution of SOM-positive neuronal cells and nerves in the human stomach and oesophagus is scarce. It is known that SOM is present in both the neuronal cells and nerve fibres localized in the gastric antrum [55]. Neurons containing SOM have been noted in both types of gastric enteric ganglia (myenteric and submucous), but more numerous SOM-positive cells have been observed in the submucous ganglia. The density of SOM-positive nerves within the antrum was considerable in the enteric ganglia, contrary to the muscular layer, where such nerves were rather rare [55]. Other studies have reported that a similar distribution of SOM-positive nerve fibres takes place in the pyloric region of the stomach [21]. Within the oesophagus, some studies have described only extremely scanty nerve fibres containing SOM in the smooth longitudinal muscle layer and the myenteric plexus [21]. Other studies, apart from nerves, have described the presence of SOM in a small number of neuronal cells in the oesophageal myenteric and submucous ganglia [22], while others still have reported that SOM-positive structures in the human oesophagus are not present [60]. It is also known that SOM is present in the nervous structures (in single neurons and relatively numerous nerve fibres) located in the wall of human gallbladder, which according to some authors, belong to the ENS [61].

### 3.4. Other Mammal Species

The presence of SOM has been also observed in the innervation of the GI tract in other mammal species. In the rat and mouse, the distribution of SOM in the ENS is generally similar to the guinea pig. This peptide has been noted in a small number of myenteric and submucous neurons (especially in the interneurons and secretomotor neurons) and nerve fibres located in the muscular and mucosal layers, as well as inside the enteric plexuses [62,63,64,65,66,67,68]. The highest density of SOM-positive nerves in the rat intestine has been described in the mucosal layer, and SOM was present especially in fibres adjacent to the endothelial cells of capillary blood vessels [69]. These nerves were often varicosities and contained synaptic vesicles, which may suggest the participation of such nerve processes in elevated SOM levels in portal blood [69]. Willard and Nishi reported the presence of SOM in the myenteric plexus of new-born rats and showed that SOM-positive cholinergic myenteric neurons cause rapid nicotinic excitatory post-synaptic potentials (EPSPs) [63]. Enteric neurons immunoreactive to SOM in the GI tract of the rat showed caudal projections in both the small and large intestine [70,71]. In the rat submucous plexus, SOM-positive neurons have been divided into two groups: those containing both CGRP and SP (60% of all neurons immunoreactive to SOM) and those containing only CGRP without SP [64]. In turn, the latest studies have shown the presence of chemokine CXCL 14 and P2X1 receptors in the SOM-positive nervous structures in the mouse digestive tract, which suggests that CXCL14 contributes to the function of somatostatin in the enteric neuron system and that ATP may participate in SOM release from the enteric neurons [72,73].

In 1984, Lolova et al. described for the first time the presence of SOM in the ENS located within the pyloric sphincter, ileum, ileocecal sphincter and proximal colon of the cat [74]. The greatest number of SOM-positive structures were found in the ileum, and the presence of SOM was more often noted in the outer and inner submucous plexuses than in the myenteric plexus, where only single SOM-positive cells were observed [74]. Moreover, nerves immunoreactive to SOM, being the processes of submucous neurons, were traced over a longer distance compared to the processes of the SOM-like immunoreactive myenteric neurons. Some SOM-positive neurons were also located outside the intramural ganglia along the nerve bundles. It should be pointed out that SOM-positive cells (in the most cases bipolar or pseudo-unipolar) were noted mainly in peripheral parts of the enteric ganglia [74].

The presence of SOM in the neuronal structures has been also studied in the canine digestive tract [75,76,77]. Contrary to the cat, neuronal cells immunoreactive to SOM were relatively numerous both in the myenteric plexus (where SOM-positive cells accounted for one-quarter of all myenteric neurons), as well as in the submucous plexuses [75,77]. Similar to other mammalian species, SOM-positive neurons located in the myenteric plexus of the small intestine showed a caudal projection [78]. In turn, nerve fibres containing SOM located in the muscular and mucosal layers, as well as inside of the enteric plexuses, were rather rare and delicate [75].

Of course, the above-mentioned species are not the only species, in which SOM has been observed in the enteric nervous structures. Some studies of this issue concern very exotic mammalian species. For example, SOM has been noted in rare nerves located in the cardiac, funding and pyloric regions of the stomach of the African elephant [79] and in the intestinal mucosal layer of the marmoset [19]. A summary of the existing observations concerning the distribution of SOM in nervous structures located in the particular parts of the GI tract in humans, domestic pigs and guinea pigs is presented in Table 1.

## 4. Distribution of SOM Receptors in the Enteric Nervous System

As mentioned above, SOM acts by means of five types of receptors (from SSTR1 to SSTR5) [3,80]. The majority of them exist in one isoform. The only exception is SSTR2, which has two isoforms: SSTR2A and SSTR2B. It is known that SOM receptors are widely distributed in the GI tract, mainly in the endocrine cells and smooth muscle cells [81,82]. The exact distribution of SOM receptors in the GI tract (similar to SOM) depends on the mammalian species and the segment of the GI tract [81]. In spite of the fact that previous studies also reported the presence of both SOM receptors protein as well as SOM receptors messenger (mRNA) in the ENS, the knowledge concerning this issue is relatively insufficient [81,83]. The majority of studies on the distribution of SOM receptors in the ENS concern SSTR1 and SSTR2.

In the rat, SSTR2 receptor was described within distinct populations of the enteric neurons localized in the intestinal myenteric and submucous plexuses, as well as in the gastric myenteric plexus [80]. Such type of receptor was also studied in the intestinal and gastric intramural nerve fibres both within and outside the enteric ganglia. First of all, SSTR2 was noted in thick bundles of nerves localized in the myenteric and mucosal layer, especially near blood vessels [80]. Fibres immunoreactive to SSTR2 were also observed in the direct vicinity of SOM–positive endocrine cells in the mucosal layer of the stomach and intestine [80]. The co-localization of SST2 and SOM in the same enteric neuronal structures were not observed, but the direct vicinity of SOM-containing intraganglionic varicose fibres and neurons immunoreactive to SSTR2 was described [80]. Moreover, studies on the distribution of SSTR1-SSTR5 mRNAs in various segments of the rat GI tract were conducted [81]. The studies showed the presence of SSTR1, SSTR2 and SSTR3 mRNAs in the neuronal cells located in the submucous and myenteric ganglia in the stomach, small intestine and colon. In turn, the presence of SSTR5 mRNA was not observed in nervous structures of any segment of the GI tract mentioned above. Moreover, some differences in the distribution of SOM receptors mRNAs, in particular parts of the rat GI tract, were detected. Namely, in the colon (contrary to other segments), the presence of SSTR4 mRNA was noted in the myenteric plexus [81].

Distribution of SOM receptors was also studied in the murine small intestine [83]. According to the study, SSTR1 was found to be present in the nerve fibres located at the base of crypts of Lieberkühn and within submucous and myenteric ganglia. 

In the majority of fibres, SSTR1 co-localizes with CGRP. Contrary to the rat, SSTR1 was not observed in intramural neuronal cells of the murine ileum [83]. In turn, SSTR2A was described both in nerve fibres and neuronal cells in the myenteric and submucous plexuses. The number of SSTR2A–positive neurons in the myenteric plexus, amounted to about 5% of the total neuronal population. SSTR2A was noted mainly in neurons showing Dogiel type-2 morphology which project to anal direction [83]. Moreover, a high degree of co-localization of SSTR2A with neuronal isoform of nitric oxide synthase in the myenteric neurons was detected. According to Van Op den Bosch et al., SSTR4 is present only in the nerve fibres (not in neuronal cells) located in the ileal myenteric and submucous plexuses, and these fibres often contain CGRP [83]. SSTR3 and SSTR5 are not present in the nervous structures in the wall of murine ileum [83]. SSTR1 and SSTR2 were also noted in the nervous structures in the human and guinea pig gastrointestinal tracts with the role of mediating inhibitory postsynaptic potentials [82,84,85].

## 5. The Plasticity of the Enteric Nervous Structures Containing Somatostatin

The term “plasticity” of the nervous system concerns adaptive changes within neuronal cells arising under the influence of modification of the environmental conditions and is intended to maintain homeostasis [86,87]. Such changes may arise from both pathological and physiological stimuli. It is relatively well known that the phenomenon of plasticity also occurs within the ENS. The enteric neurons may undergo modification under (among others) the influence of gastrointestinal or systemic diseases, metabolic disorders, toxic substances in food and mechanical injury of nerves supplying the intestine [9,12,15,88]. Changes in the ENS may also be connected with physiological stimuli, involving the growth, changes in diet and maturation and ageing of the body [89]. Adaptive modifications in the enteric neurons are multidirectional and very differentiated. They may be manifested in changes in the structure of the ENS (especially in modifications of number, size and shape of neurons), as well as alterations in the function and electrophysiological properties of the enteric neurons [90]. Nevertheless, the most visible adaptive changes in the ENS, both under physiological and pathological stimuli, concern the modification of neurochemical characterization of the enteric neurons [9,12,15,89,90]. 

Previous studies have shown that the number of SOM-positive enteric nervous structures is also subject to change in response to various stimuli. The character and intensity of these changes clearly depend on the acting stimulus, segment of the digestive tract, part of the ENS and mammalian species studied. The first publication concerning a decrease of SOM concentration in the wall of the guinea pig ileum after extrinsic innervation was published in 1977, shortly after the discovery of this peptide [26]. In subsequent studies, a decrease in the number of SOM-positive enteric neurons and/or nerve fibres was observed in the rat gastrointestinal tract during experimental diabetes [91,92] and in the rat jejunum after administration of benzalkonium chloride [93]. Similar changes have been noted in the human large intestine during colonic cancer (changes were observed in the number of SOM-positive cells in the enteric plexuses, but not in the number of fibres containing SOM) [53]. In turn, in the porcine ascending colon during experimental *Bacteroides fragilis* infection, the decrease in the number of SOM–immunoreactive cells–was accompanied by an increase in the density of SOM-positive intraganglionic nerves [47].

Various changes in the number of SOM-positive enteric neurons and nerves, clearly dependent on the part of the ENS and acting stimulus, were described in the porcine descending colon [17]. It has been shown that even the same stimulus may cause various changes in various parts of the ENS. Such a situation has been noted in the case of chemically-induced colitis, which caused an increase in the number of SOM-positive nervous structures within the ISP and muscular layer with a simultaneous decrease in the OSP and mucosal layer [17]. A similar phenomenon accompanied the proliferative enteropathy, during which a reduction in the number of SOM-positive neurons and nerve fibres was observed in the MP, mucosal and muscular layers, and an increase in the percentage of such cells in the ISP was detected [17]. Other changes under the same pathological state (proliferative enteropathy) were observed by Pidsudko et al. in the porcine small intestine (ileum) [20]. These authors observed a several-fold increase in the number of SOM-positive neuronal cells in all types of the enteric plexuses. The increase in the number of the enteric neuronal structures containing SOM was also noted under the influence of physiological stimuli, e.g., in the embryo-foetal development in the guinea pig [94].

Interestingly, the previous studies reported more than once that strong pathological stimuli, which are known to influence the neurochemical characterization of the enteric neurons, have no effect on the number of SOM-immunoreactive nervous structures in the GI tract. Such a situation has been noted, among others, in the human colon during Hirschsprung disease [54], within the human pylorus during hypertrophic pyloric stenosis [21], and after massive bowel resection in the rat small intestine, although in the latter case a clear increase in the size of SOM-positive enteric neurons was reported [95]. A summary of the existing observations concerning the changes in the number of SOM-positive nervous structures in the wall of the GI tract under various stimuli is presented in Table 2.

## 6. SOM in the Extrinsic Innervation of the Gastrointestinal (GI) Tract

**Figure 3:**
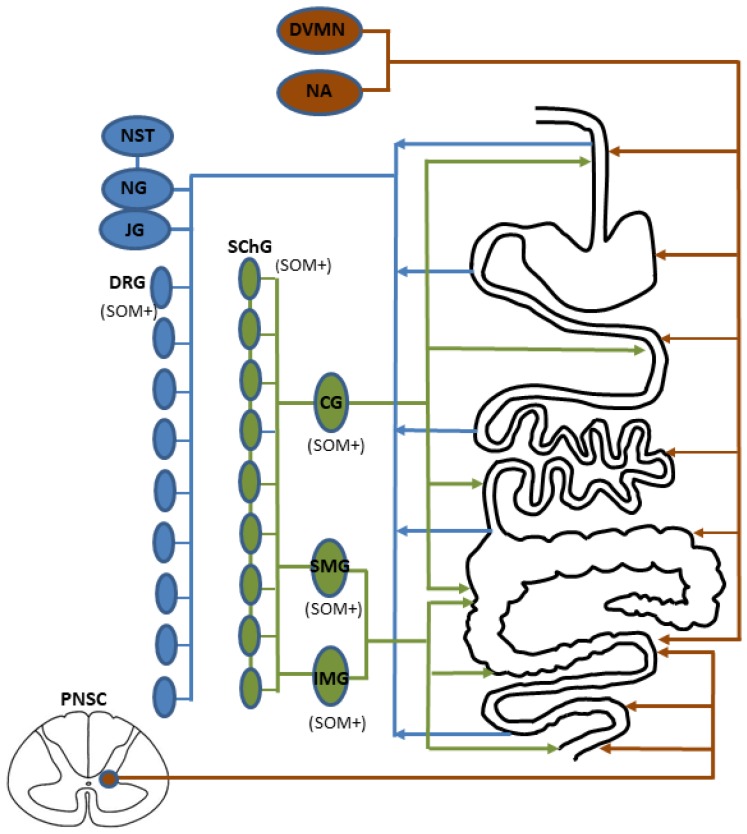
Organization of the extrinsic innervation of the gastrointestinal tract: blue–sensory innervation, green–sympathetic innervation, brown–parasympathetic innervation, DVMN = dorsal vagal motor nucleus, NA = nucleus ambiguous, PNSC = parasympathetic nuclei of the sacral spinal cord, NST = nucleus of the solitary tract, NG = nodose ganglion, JG = jugular ganglion, DRG = dorsal root ganglia, SChG = ganglia of the sympathetic trunk, CG = celiac ganglion, SMG = superior mesenteric ganglion, IMG = inferior mesenteric ganglion. Structures, where SOM was observed in neurons supplying the gastrointestinal tract are marked by “(SOM+)”.

Apart from the ENS, the functions of the GI tract are regulated by extrinsic innervation, in which three main parts can be distinguished: afferent sensory, sympathetic, and parasympathetic neuronal cells (Figure 3) [96,97,98,99]. 

Sensory neurons conduct sensory and pain stimuli from the GI tract to the central nervous system. It has been shown that the perikarya of these neurons are located in the dorsal root ganglia (DRG) and in the sensory ganglia of the vagal nerve, such as jugular and nodose ganglia [97,100,101]. Moreover, it is known that the localization of sensory neurons depends on the segment of the GI tract they innervate. Neurons located in the DRG of thoracic neuromeres innervate the stomach and duodenum, DRG in lumbar neuromeres innervate the jejunum and ileum, and DRG in sacral neuromeres innervate the colon [97,100,101]. In turn, neurons located in the sensory ganglia of the vagal nerve supply the GI tract from the oesophagus to the proximal colon.

The next part of the extrinsic innervation of the GI tract is parasympathetic innervation. Cell bodies of parasympathetic neurons supplying the GI tract are located in dorsal motor nucleus and nucleus ambiguous of the vagal nerve (these neurons supply the GI tract from the oesophagus to the proximal colon), as well as in parasympathetic nuclei of the sacral spinal cord (they innervate the distal colon and rectum) [99,102,103,104]. In turn, the cell bodies of sympathetic neurons supplying the cranial part of the GI tract (from oesophagus to proximal colon) are localized in celiac ganglia, and those innervating distal colon and rectum are placed in the superior and inferior mesenteric ganglia [105,106,107]. Moreover, it is known that sympathetic neurons supplying the GI tract are also present in the sympathetic chain [98]. 

It should be pointed out that knowledge concerning the occurrence of SOM in the extrinsic innervation of the GI tract is rather scarce. The greatest number of studies apply to the distribution of SOM in the intestinal sympathetic innervation. The presence of SOM in a relatively large number of neuronal cells (from 25% to 60% of the total neuronal population) located in the coeliaco-superior mesenteric ganglion complex of the guinea pig was first described in the 1970s [108]. The first description of SOM-positive extrinsic nerves (which were the processes of neurons located in the coeliaco-mesenteric ganglia) located in the wall of the guinea pig small intestine and, first of all, supplying the submucous plexus and mucosal layer, was made by Costa and Furness [109]. SOM-positive sympathetic extrinsic nerves have been also described in the rat small intestine [92]. Neurons supplying the rat GI tract containing SOM were detected in dorsal root ganglia, but not in the nodose ganglia [110]. The presence of SOM in DRG neurons supplying the GI tract in the rat was confirmed by Traub et al., who observed that SOM-positive neurons amounted to 20%–30% of all neuronal cells located in the lumbar and sacral dorsal root ganglia [111].

Nevertheless, the most thorough investigations concerning the distribution of SOM in the extrinsic gastrointestinal innervation have been conducted on the domestic pig, which may be the best animal model of human gastrointestinal innervation [42]. In the domestic pig, SOM has been observed in neurons supplying the descending colon and located in the inferior mesenteric ganglia (IMG) [105,106]. Such neurons under physiological conditions amounted to about 12% of the total IMG neuronal cells supplying the colon and often showed the co-localization of SOM and GAL. Moreover, the number of these neuronal cells clearly decreased during pathological states concerning the descending colon, including chemically-induced colitis and proliferative enteropathy [105,106]. In turn, the neurons containing SOM and supplying the stomach have been noted in the coeliac-superior mesenteric ganglion (CSMG) complexes [107]. Such neurons amounted to about 15% of all CSMG neuronal cells innervating the prepyloric region of the stomach, and their number increased after acetylsalicylic acid administration and partial resection of the stomach, as well as during experimental hyperacidity [107]. An interesting situation was observed in the ganglia of the sympathetic chain. Under physiological conditions, SOM-positive neuronal cells supplying the descending colon were not observed [90,112], but after injury to the nerves supplying the colon, such neurons appeared in the lumbar part of the sympathetic chain, and their number amounted to 7% of all neuronal cells supplying the descending colon [90]. Apart from the sympathetic extrinsic innervation of the porcine digestive tract, SOM in this species was also noted in a small population of sensory neurons supplying the descending colon and located in the lumbar and sacral dorsal root ganglia [113].

## 7. Functions of Somatostatin in the Enteric Nervous System

The functions of SOM within the ENS have been the objective of studies from the 1970s, but until now, all aspects connected with SOM activity in the enteric neurons have not been fully elucidated.

The first papers concerning the functions of SOM in the ENS, published in the 1970s and 1980s, showed that SOM inhibits the release of acetylcholine from neurons located in the myenteric plexus, and, therefore, it may contribute to the inhibition of the intestinal muscle contraction [114,115]. Simultaneously, it has been noted that this inhibitory effects of SOM did not depend on the presence of extracellular calcium ions but was probably connected with membrane hyperpolarization [116]. Subsequent studies confirmed that SOM plays inhibitory functions in the enteric interneurons and is involved in the descending inhibitory reflex of peristalsis [26,27]. The inhibitory effect of SOM on the interneurons was confirmed by the fact that the direct effects of SOM on the smooth intestinal muscles was observed [28]. It has also been shown that SOM in vitro inhibits peristalsis [117], and in vivo, inhibits the migrating myoelectric complex, antagonizes the intestinal propulsion and leads to deceleration gastric emptying [118,119]. Other studies have shown that SOM may induce both hyperpolarization and depolarization of the enteric neurons, and the character of SOM activity depends on the method of SOM administration and electrophysiological properties of the neurons treated with this peptide [120]. SOM-induced depolarization was connected with an increase in membrane resistance, as observed by the change in the amplitude of anelectrotonic potentials, and the hyperpolarization caused by SOM resulted from a reduction in the amplitude of anelectrotonic potentials [120]. Other studies have noted that SOM had no effects on colonic contraction, where the muscles are at rest, but caused a very small reduction in the amplitude of the contraction of the colonic smooth muscles in response to electrical stimulation [36,121]. 

Studies on the influence of SOM on acetylcholine release by the myenteric neurons isolated from the guinea pig intestine were continued by Yau et al. [122]. They observed that SOM inhibits acetylcholine release both in neurons at rest and under electrical stimulation. This inhibition depended on the dose of SOM but was never complete. There was a 40% fraction of total release of acetylcholine which remained resistant to somatostatin. Subsequent experiments reported that SOM not only inhibits acetylcholine release, but can also selectively modify the effects of other peptides affecting the myenteric neurons, including [123]. However, the influence of SOM on SP-induced changes in the contractility of ileal muscles was not observed. Other studies have confirmed that the inhibitory effects of SOM on acetylcholine release by the myenteric neurons are based on other mechanisms than processes in which met-enkephalin is involved [124]. So, these investigations describe the two main kinds (SOM-positive and Met-enkephalin-positive) of inhibitory neurons, which have distinct neuromodulatory functions in the cholinergic pathways within the ENS. It has also been shown that SOM-induced relaxation of the intestinal muscles is connected with the pre-synaptic inhibition of acetylcholine release by different mechanisms [125,126]. The main mechanism of this SOM activity results from the inhibitory effects on 3’,5’-cyclic adenosine monophosphate [125,126]. A similar activity of SOM was observed in the submucous plexuses, where in the guinea pig intestine SOM caused the hyperpolarization of about 90% of the total neuronal population [127]. This phenomenon is connected with the influence of potassium channels and enhancing the potassium in neurons [127,128]. It leads to the inhibition of the secretory activity of the intestinal mucosal layer, as well as enhancing the absorptive activity of the intestine [127].

Apart from the involvement of SOM in the inhibition of gastrointestinal motility and secretory activity, SOM in the ENS may also play various other functions. As mentioned above, previous studies have reported the presence of SOM both in the intrinsic primary afferent neurons located in the wall of the intestine [18,58] and in the extrinsic sensory neurons supplying the GI tract [110,113]. This fact strongly suggests the participation of SOM in sensory and pain stimuli conduction both within the ENS and from the GI tract to the central nervous system. Moreover, SOM not only affects acetylcholine release, as described above, but also participates in modulation of the release of other neuropeptides. An example of such activity is the inhibitory feedback between vasoactive intestinal polypeptide and SOM observed in the intestinal myenteric plexus [129]. In this inhibitory feedback, SOM induces an increase in the secretion of VIP which, in turn, causes a decrease in somatostatin release from interneurons.

Other studies have reported that SOM may participate in the regulation of the intestinal blood flow [129]. In turn, the high density of somatostatin receptors expressed in the intestinal blood vessels, especially during inflammatory bowel disease, suggests that this activity of SOM is very important during gastrointestinal pathological states [130]. The involvement of SOM in pathological mechanisms has been also confirmed by numerous studies describing the changes in the number of SOM-positive nervous structures in both the ENS and in the extrinsic intestinal innervation under the influence of various stimuli [17,47,105,106,107]. These changes may be connected with the fact that SOM (considered to be a strong anti-inflammatory factor) stimulates B-lymphoblast proliferation with the simultaneous induction of immunoglobulin synthesis, reduces the activity of T lymphocytes and granulocyte proliferation and inhibits the synthesis of proinflammatory cytokines [131,132,133,134]. SOM also participates in the so-called “somatostatinergic anti-inflammatory loop”, in which damaging stimuli affect the peptidergic nerve endings and cause the release of substance P and/or calcitonin gene-related peptides known as pro-inflammatory agents [134]. These agents induce local neurogenic inflammation which, in turn, causes the release of SOM from the same peptidergic nerves. SOM stops the release of substance P and calcitonin gene-related peptide and inhibits inflammatory processes.

The above-mentioned anti-inflammatory properties of SOM have led to this peptide and its analogues (especially octreotide) becoming the subject of studies aimed at the eventual application of these substances during the treatment of intestinal inflammatory processes. Previous studies have shown that octreotide inhibits the feeling of pain during inflammatory processes in the intestine [135]. It also reduces hyperaemia, bowel secretion and intraluminal pressure (anti-diarrheal activity) and has potential immunomodulatory effects [136,137,138,139]. During studies on experimentally-induced inflammatory processes, it has also been shown that octreotide effectively protects the intestinal mucosa against damage [140]. Due to its activity, octreotide may be used in the treatment of inflammatory bowel disease, Crohn’s disease, intestinal tumours, as well as in patients with intestinal damage after ischemia [141,142,143,144,145].

## 8. Conclusions

Previous studies have indicated that SOM is an important neuronal factor participating in the regulation of GI tract functions. The presence of this peptide has been noted in numerous mammalian species, both in the enteric nervous system of various segments of the digestive tract, as well as in the extrinsic innervation of the stomach and intestine. The exact number of SOM-positive neuronal structures in the ENS clearly depends on the mammalian species and segment of the GI tract. SOM located in the ENS may play multidirectional functions. Among others, it inhibits intestinal motility and secretory activity of the mucosal layer, participates in the sensory and pain stimuli conduction, and regulates blood flow in the wall of the GI tract. The major functions of SOM are connected with pathological changes within the intestine and the anti-inflammatory properties of this peptide have led to interest in SOM analogues being used in the treatment of various intestinal diseases. Nevertheless, the elucidation of all aspects of the role of SOM in the ENS, as well as the suitability of SOM analogues in the treatment of particular intestinal diseases, requires further studies.

## Figures and Tables

**Table 1:** Distribution of somatostatin in the nervous structures located in the wall of the gastrointestinal tract in the human, domestic pig and guinea pig.

Part of the Gastrointestinal Tract	Localization of Somatostatin	References
**Human**		
oesophagus	intramural nerve fibres, neuronal cells located in the myenteric plexus and submucous ganglia,	[21,22]
stomach	neuronal cells in the myenteric plexus and submucous ganglia located in the pyloric region and antrum, intraganglionic nerve fibres, nerves in the muscular and mucosal layers nerves in the muscularis mucosae	[19,21,55]
small intestine	neuronal cells located in the myenteric plexus, plexus submucous externus and plexus submucous internus, intraganglionic nerves nerves in the muscular and mucosal layers	[18,19,54,57,58]
large intestine	neuronal cells located in the myenteric plexus, plexus submucous externus and plexus submucous internus, intraganglionic nerve fibres in all types of enteric plexuses, nerve fibres in the muscular and mucosal layers,	[18,19,53,59]
**Domestic pig**		
oesophagus	rare intramural nerve fibres in the muscular and mucosal layer,	[52]
stomach	single neurons in the submucous ganglia, rare nerves in the muscular and mucosal layers,	[51]
small intestine	- neuronal cells in the myenteric, outer submucous and inner submucous plexuses, intraganglionic nerve fibres, nerve fibres in the muscular and mucosal layers,	[20,49,50]
large intestine	neuronal cells located in the myenteric plexus, plexus submucous externus and plexus submucous internus, intraganglionic nerve fibres in all types of enteric plexuses, nerve fibres in the muscular and mucosal layers,	[17,47,48]
**Guinea pig**		
oesophagus	rare nerve fibres in muscularis mucosae and myenteric plexus	[19]
stomach	neuronal cells and nerve fibres in the myenteric plexus nerve fibres in the muscular layer nerve fibres in the mucosal layer	[38,39,40,41]
small intestine	neuronal cells in the myenteric and submucous plexus nerves in the enteric ganglia, nerve fibres in the mucosal and myenteric layers	[16,26,27,28,29,30,31,32,33,34]
large intestine	neuronal cells located in the myenteric and submucous plexus, intraganglionic nerve fibres, nerve fibres located in the muscular and mucosal layers,	[16,27,35,36,37]

**Table 2:** The changes in the number of somatostatin-positive (SOM+) nervous structures in the wall of the digestive tract under physiological and pathological stimuli: ⭡ an increase in the number of SOM+ nervous structures, ⭣ a decrease in the number of SOM+ nervous structures, ⭡⭣ various changes depending on the part of the enteric nervous system, ⭤ changes were not observed.

Stimulus	Character of Changes	References
**Human**		
Hirschsprung disease	⭤	[54]
Hypertrophic pyloric stenosis	⭤	[21]
Colonic cancer	⭣	[53]
**Guinea pig**		
Embryo-foetal development	⭡	[93]
Extrinsic denervation	⭣	[26]
**Rat**		
Bowel resection	⭤	[95]
Experimental diabetes	⭣	[91,92]
Benzalkonium chloride administration	⭣	[92]
**Domestic pig**		
Experimental Bacteroides fragilis infection	⭡⭣	[47]
Proliferative enteropathy in the ileum	⭡	[20]
Proliferative enteropathy in the colon	⭡⭣	[17]
Extrinsic denervation (colon)	⭡⭣	[17]
Chemically induced colitis	⭡⭣	[17]
Swine dysentery (stomach)	⭤	[51]
